# Comparison of Management and Outcomes of Hip Fractures Among Low- and High-Income Patients in Six High-Income Countries

**DOI:** 10.1007/s11606-024-09274-9

**Published:** 2024-12-20

**Authors:** Nicole Huang, Laura A. Hatfield, Saeed Al-Azazi, Pieter Bakx, Amitava Banerjee, Nitzan Burrack, Yu-Chin Chen, Christina Fu, Carlos Godoy Junior, Renaud Heine, Dennis T. Ko, Lisa M. Lix, Victor Novack, Laura Pasea, Feng Qiu, Bheeshma Ravi, Therese A. Stukel, Carin Uyl-de Groot, Peter Cram, Bruce E. Landon

**Affiliations:** 1 Institute of Hospital and Health Care Administration, National Yang Ming Chiao Tung University, Taipei, Taiwan; 2 Department of Health Care Policy, Harvard Medical School, Boston, MA USA; 3 Department of Community Health Sciences, University of Manitoba, Winnipeg, Manitoba Canada; 4 George & Fay Yee Centre for Healthcare Innovation, University of Manitoba, Winnipeg, Manitoba Canada; 5 Erasmus School of Health Policy & Management, Erasmus University, Rotterdam, the Netherlands; 6 Institute of Health Informatics, University College London, London, England; 7 Department of Cardiology, University College London Hospitals, London, England; 8 Clinical Research Center, Soroka University Medical Center, Faculty of Health Sciences, Ben Gurion University of the Negev, Beersheba, Israel; 9 Schulich Heart Program, Sunnybrook Health Sciences Centre, Sunnybrook Research Institute, Toronto, ON Canada; 10 ICES, Toronto, ON Canada; 11 Faculty of Medicine, University of Toronto, Toronto, ON Canada; 12 Division of Orthopaedic Surgery, Department of Surgery, University of Toronto, Toronto, Ontario Canada; 13 Division of Orthopaedic Surgery, Sunnybrook Health Sciences Centre, Toronto, Ontario Canada; 14 Institute of Health Policy, Management and Evaluation, University of Toronto, Toronto, Canada; 15 Department of Medicine, UTMB, Galveston, TX USA; 16 Division of General Medicine, Beth Israel Deaconess Medical Center, Boston, MA USA

**Keywords:** Hip fracture, International comparisons, Disparities in healthcare

## Abstract

**Background:**

There is a perception that income-based disparities are present in most countries but may differ in magnitude. However, there are few international comparisons that describe income-based disparities across countries and none that focus on hip fractures.

**Objective:**

To compare treatment patterns and outcomes of high- and low-income older adults hospitalized with hip fracture across six high-income countries.

**Design:**

Retrospective serial cross-sectional cohort study.

**Participants:**

Adults aged ≥ 66 years hospitalized with hip fracture from 2013 to 2019 in Canada, England, Israel, the Netherlands, Taiwan, and the USA using population-representative patient-level administrative data.

**Main Measures:**

Older adults in the top and bottom income quintiles within countries were compared on 30-day and 1-year mortality, treatment approaches, hospital length of stay (LOS), 30-day readmission rates, time to surgery, and discharge disposition.

**Key Results:**

Annual age- and sex-standardized incidence rates of hip fracture were higher for low- than for high-income populations in all countries except in the USA. In all countries, adjusted 1-year mortality was lower for high-income than low-income patients, with the largest difference in Israel (− 10.0 percentage points [95% confidence interval [CI], − 15.2 to − 4.8 percentage points]). Across countries, utilization of total hip arthroplasty was 0.1 (95% CI, 0.0–0.2 percentage points) to 6.9 percentage points (95% CI, 4.6–9.2 percentage points) higher among high- vs. low-income populations. With few exceptions, LOS, adjusted 30-day readmission rate, and time to surgery were shorter and lower for high-income patients.

**Conclusions:**

Income-based disparities in treatments and outcomes for older adults hospitalized for hip fractures differed in magnitude, but were present in all six high-income countries. Defying our expectations, the USA did not have consistently larger disparities than other countries suggesting that the impacts of poverty exist in vastly different healthcare systems and transcend geopolitical borders.

**Supplementary Information:**

The online version contains supplementary material available at 10.1007/s11606-024-09274-9.

## INTRODUCTION

Though disparities in care have been a significant concern in most high-income countries, there has been a lack of appreciation of whether these disparities are uniquely related to a country’s health system structure (aka, “American exceptionalism”) or more universal in nature.^1–3^ International comparison of treatment patterns and outcomes of specific diseases can provide policy makers and healthcare leaders useful insights to identify strengths and weaknesses in the financing and organization of their healthcare systems for further improvement and policy development.^4–6^ Similarly, international comparisons can help to draw attention to common challenges facing clinicians around the world in treating disadvantaged patient groups.^4,6,7^

Prior comparative work typically has focused on aggregated performance of healthcare systems; while important, these comparisons offer limited understanding of country performance in income-based inequities.^4–6,8^ Although researchers have used surveys and registry-based mortality data to compare income disparities in several domains of care,^9–13^ the use of routinely collected patient-level data from population-representative sources allows for detailed analyses of income-based disparities in the management and outcomes of specific diseases by focusing on a homogeneous clinical population in each country.^5,14,15^ To date, international comparisons have been limited to studies of access to general care, cardiovascular conditions, and two-country comparisons between the USA and Canada or England.^1,7,16,17^ Thus, there is a need to expand the focus of this research to examine additional conditions.

Hip fracture is a costly and prevalent condition among the elderly. Despite advances in surgical treatments and postoperative management, hip fracture causes significant morbidity and mortality in older adults.^18,19^ Hip fracture patients are almost always hospitalized, thus minimizing selection bias (due to elective admission decisions) which could confound between country comparisons focusing on hospitalized patients. In a prior paper, we found that income-based disparities in treatments and outcomes of myocardial infarction were consistent across six different high-income countries, but at present, it is unknown if income-based disparities extent to hip fractures. For people with hip fractures, there are several different surgical procedures as well as non-operative management that can be used^20–22^ and these treatment options differ significantly in cost and complexity. Consequently, even for countries offering universal health insurance, there are concerns that economically disadvantaged patients would be treated using different approaches and potentially have worse outcomes. In this study, we compared treatment processes and outcomes between hip fracture patients living in low- and high-income neighborhoods using population-representative patient-level data from the six high-income countries (Canada, England, Israel, the Netherlands, Taiwan, and the USA) participating in the International Health Systems Research Collaborative (IHSRC: https://projects.iq.harvard.edu/ihsrc/people). ^7,14,23^ These six high-income countries were compared because they all have relatively comparable population-representative data available for research and offer universal health coverage to their older populations, but differ widely with respect to how healthcare system is organized, reimbursed, and financed.

## MATERIALS AND METHODS

### Data and Study Population

We identified adults aged 66 years and older who were newly admitted to a hospital with a primary diagnosis of hip fracture between January 1, 2013, and December 31, 2019 (December 31, 2018 in England), in six IHSRC countries (Canada is represented by the provinces of Manitoba and Ontario). Each country used standardized coding based on International Classification of Diseases, Ninth/Tenth Revision (ICD-9/10) to identify patients hospitalized with hip fracture from population-representative claims data, with a few minor country-specific adaptations (see eTable I and eTable II).^7,14,23–30^

We excluded patients with high-energy hip fracture or with a hip fracture admission in the 180 days prior to the index admission. We also excluded small numbers of patients with missing values of age or sex, residences outside of the jurisdiction of admission, and who lacked a year of preadmission or postadmission follow-up data (except in the case of death). For patients who were transferred between hospitals, admission records were linked and the complete episodes of care from initial admission to final discharge was evaluated. The same inclusion and exclusion criteria were applied in the same order (see eTable III).

We identified comorbid conditions using the Manitoba adaptation of the Elixhauser Comorbidity Index from the index admission and all prior admission records in the prior year.^31^ Since Israel’s medical record systems integrate both hospital and ambulatory care visits, comorbid conditions identified included those recorded in ambulatory care settings. In the Netherlands, comorbidities were unavailable from inpatient claims, so we used medications related to specific chronic conditions to capture comorbidities.^7,14,23^

### Ascertaining Income Status

With some modifications, high- and low-income patients were defined based on residing in areas in the top or bottom 20% of the income distribution, where the distributions were defined within relevant regions for each country (see eTable IV). In Israel and England, the quintiles were defined at a national level, but also verified in sensitivity analyses using smaller geographic areas. Due to differences in population size and geographic characteristics across the six countries, regional sizes used to categorize income status differ across countries. This approach may lead to potential misclassification at the individual level and variability in the misclassification may depend on the income mixing within geographic units of each country.^7^ In the Netherlands, we used individual household income.

### Outcomes

First, we estimated age-, sex-, and comorbidity-adjusted mortality within 30 days and 1 year of the index admission. Mortality data included deaths both within and outside hospitals. Second, we calculated the proportion of hip fracture patients receiving each of four treatment modalities (total hip arthroplasty [THA], hemiarthroplasty [HA], internal fixation [IF], or non-operative management) during the index admission. Patients without any record of surgical procedure codes were assumed to have received non-operative management.^23,32^ ICD-9 and ICD-10 codes were used to identify procedures, with some country-specific variations (see eTable II). Third, we compared hospital length of stay (LOS) and readmission within 30 days of discharge, among those who were discharged alive. Finally, discharge disposition (home versus elsewhere) and days from hospital admission to surgery for those who received surgery were assessed in the countries where these data were available.

### Statistical Analyses

Our analyses focus on differences between high- and low-income patients within each country, which we defined based on area-level-measures (for ease of presentation, we refer to these populations as “high-income” and “low-income”). First, we computed annual hip fracture incidence, standardized to the age and sex distribution of low-income patients in each country (with the exception of England where we lacked information on denominator populations by income). Second, we compared 30-day mortality, 1-year mortality, and 30-day readmission rates between high- and low-income patients within each country after adjusting for age, sex, and comorbidities. Coding of comorbid conditions differs substantially across countries, which precludes adjustment for comorbid conditions in between-country comparisons, but does not bias within-country comparisons.^7,14^ Age- and sex-standardized mortality and readmission are available in the Appendix (eFigures 1 and 2). Third, we calculated age- and sex-standardized rates of treatment modality during the index admission, LOS, discharge disposition, and days from admission to surgery within each country. We conducted the analyses by pooling all years of data.

The analyses were conducted by local research teams in each country, and ethics approval was obtained locally in each jurisdiction. SAS (Canada, Taiwan, and USA) and R (England, Israel, and the Netherlands) were used to conduct the analyses.

## RESULTS

Our study included a total of 1,407,385 older patients hospitalized for hip fracture. Hip fracture hospitalizations in the study sample ranged from 20,805 in Israel to 1,045,854 in the USA. Age- and sex-standardized incidence rates of hip fracture were higher for low- than for high-income populations in all countries except in the USA (Table 1). The mean age of the hip fracture cohorts was approximately 84 years in most countries, but somewhat younger in Taiwan. Low-income populations were younger than high-income populations in four countries. Patients in Israel had the largest age gradient between the income strata, with low-income patients being 4·4 years younger than high-income. Though over 61% of hip fracture patients were female, there were no clear differences in the sex distribution for high- vs. low-income across countries. Comorbidities were generally similar, with few exceptions (e.g., diabetes in Israel was 242 out of 515 [47%] vs. 511 out of 2240 [23%]) (see eTable V). The ratio of income among high- versus low-income patients ranged from 1.4 in Taiwan to 4.6 in Israel.

**Table 1:** Study Population by Jurisdiction for Income Quintiles Across All Study Years (2013–2019)

	Canada		England^a^		Israel		Netherlands		Taiwan		USA	
	1st (poorest) quintile (*n* = 18,540)	5th (wealthiest) quintile (*n* = 12,110)	1st (poorest) quintile (*n* = 7847)	5th (wealthiest) quintile (*n* = 11,993)	1st (poorest) quintile (*n* = 515)	5th (wealthiest) quintile (*n* = 2240)	1st (poorest) quintile (*n* = 32,509)	5th (wealthiest) quintile (*n* = 9668)	1st (poorest) quintile (*n* = 27,072)	5th (wealthiest) quintile (*n* = 23,165)	1st (poorest) quintile (*n* = 204,053)	5th (wealthiest) quintile (*n* = 205,574)
Hip fix incidence per 1000^b^	5.5	4.7	NA	NA	5.3	3.9	8.4	4.9	11.6	10.2	4.8	5.2
Demographics												
Age	83.7	83.9	83.7	85.2	79.9	84.3	84.6	82.3	81.9	81.6	83.6	84.3
Female (%)	71.6	71.1	71.2	72.4	61.6	67.4	76.6	66.3	66.5	66.8	71.8	72
Comorbidities^c^												
Hypertension	28.7	26.4	60.0	58.8	64.3	57.1	27.3	22.7	45.8	43.8	60.4	59.0
Diabetes	7.4	6.7	19.2	14.3	47.0	22.8	18.0	11.3	27.2	26.2	19.1	14.5
Congestive heart failure	4.8	4.1	14.5	11.9	15.5	12.3	0.9	0.7	6.8	6.1	22.7	20.6
Hypothyroidism	3.0	2.9	11.7	11.6	7.0	15.9	2.5	2.1	0.4	0.5	24.3	26.8
Income metrics												
Average income, local currency	41,982^d^	115,498^d^	27,913^e^	41,116^e^	93,912^f^	427,008^f^	17,401^ g^	73,110^ g^	537,600^ h^	740,400^ h^	37,559^i^	86,802^i^
Average income, USD	31,646^d^	87,062^d^	35,676^e^	52,550^e^	26,145^f^	118,879^f^	20,521^ g^	86,219^ g^	17,418^ h^	23,989^ h^	37,559	86,802
Ratio of average incomes^j^	1.0	2.8	1.0	1.5	1.0	4.6	1.0	4.2	1.0	1.4	1.0	2.3
Gini index of income inequality	32.5^ k^		32.6^ l^		38.6^ m^		26.0^n^		34.1^o^		39.0^p^	

^a^England reflects cases from 2013 to 2018

^b^Age- and sex-standardized number of admissions per year during the study period

^c^Listed comorbidities were selected for parsimony and relevance to hip fracture. Additional details are in eTable 4 in Supplement 1

^d^Median neighborhood income of sample, Canadian Dollars. Average Canadian Dollar to US Dollar exchange rate over 2019: 0.7538 USD

^e^Mean net income by SES quintile, not based on the study sample, British pounds. Data from English Indexes of Deprivation 2019 and the UK Office of National Statistics. Average British pound to US Dollar exchange rate over 2019: 1.2781 USD

^f^Mean net household income by SES quintile for 2018 only, not based on study sample, Shekels. Average Shekel to US Dollar exchange rate over 2018: 0.2784 USD

^g^Median household income of sample, Euros. Average Euro to US Dollar exchange rate over 2019: 1.1793 USD

^h^Median household income of sample, New Taiwan Dollars. Average New Taiwan Dollar to US Dollar exchange rate over 2019: 0.0323 USD

^i^Median neighborhood income of sample, US dollars. Data from US Census

^j^The income quintiles empirically defined differ from other available reports of income disparities for at least 2 different reasons. First, use of area levels measures serves to dampen the disparity between the highest and lowest quintile. Second, this study focuses on an older population and for those at the high end of the income spectrum, the use of income may underestimate differences in underlying wealth. The reference is the poorest quintile

^k^As of 2018, World Bank. Higher scores indicate greater inequality

^l^As of 2020, World Bank. Higher scores indicate greater inequality

^m^As of 2018, World Bank. Higher scores indicate greater inequality

^n^As of 2020, World Bank. Higher scores indicate greater inequality

^o^As of 2017, Statista. Higher scores indicate greater inequality

^p^As of 2020, World Bank. Higher scores indicate greater inequality

### Mortality

**Figure 1:**
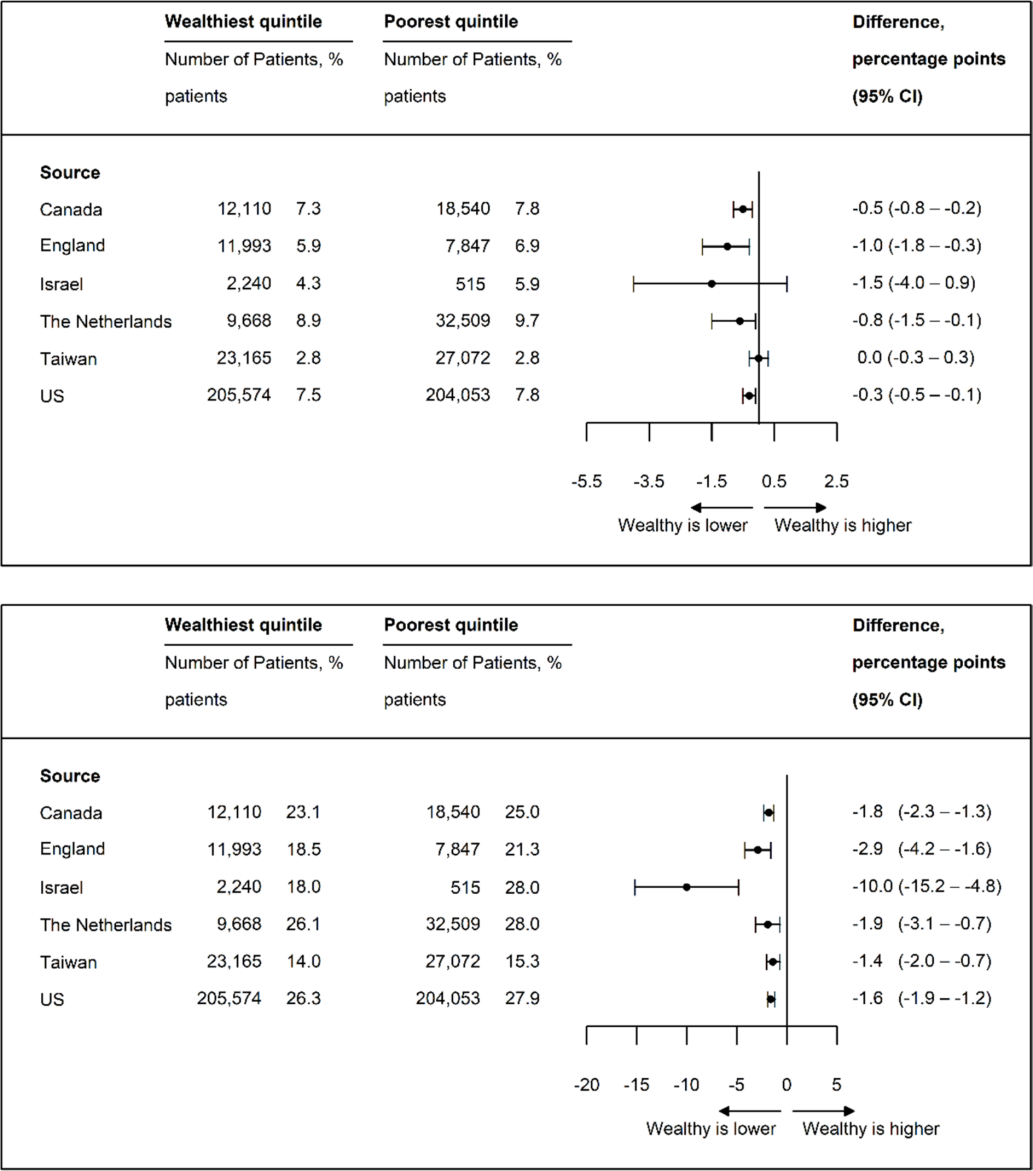
**a** Adjusted 30-day mortality rate*. **b** Adjusted 1-year mortality rate*. Adjusted for age, sex, and comorbidity.

Across all countries, high-income patients had lower adjusted 30-day and 1-year mortality rates than low-income patients (Fig. 1a and b). Israel had the largest difference between high- and low-income patients in 1-year mortality (− 10.0 percentage points [pp]; 95% confidence interval [CI], − 15.2 to − 4.8 pp), followed by England (− 2.9 pp; 95% CI, − 4.2 to − 1.6 pp). The smallest differences were in Taiwan (− 1.4 pp; 95% CI, − 2.0 to − 0.7 pp) and the USA (− 1.6 pp; 95% CI, − 1.9 to − 1.2 pp).

### Surgical Approach

**Figure 2:**
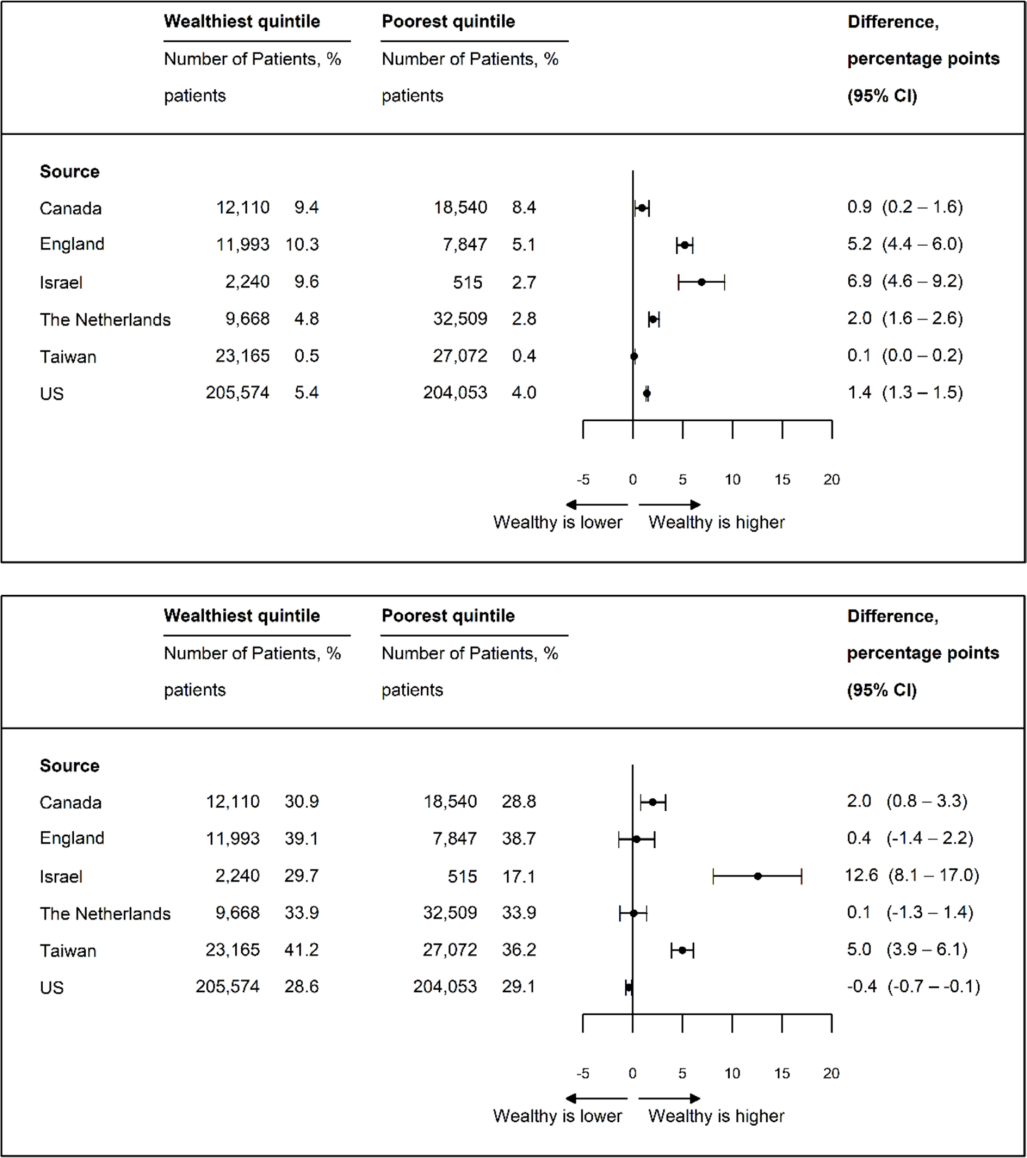

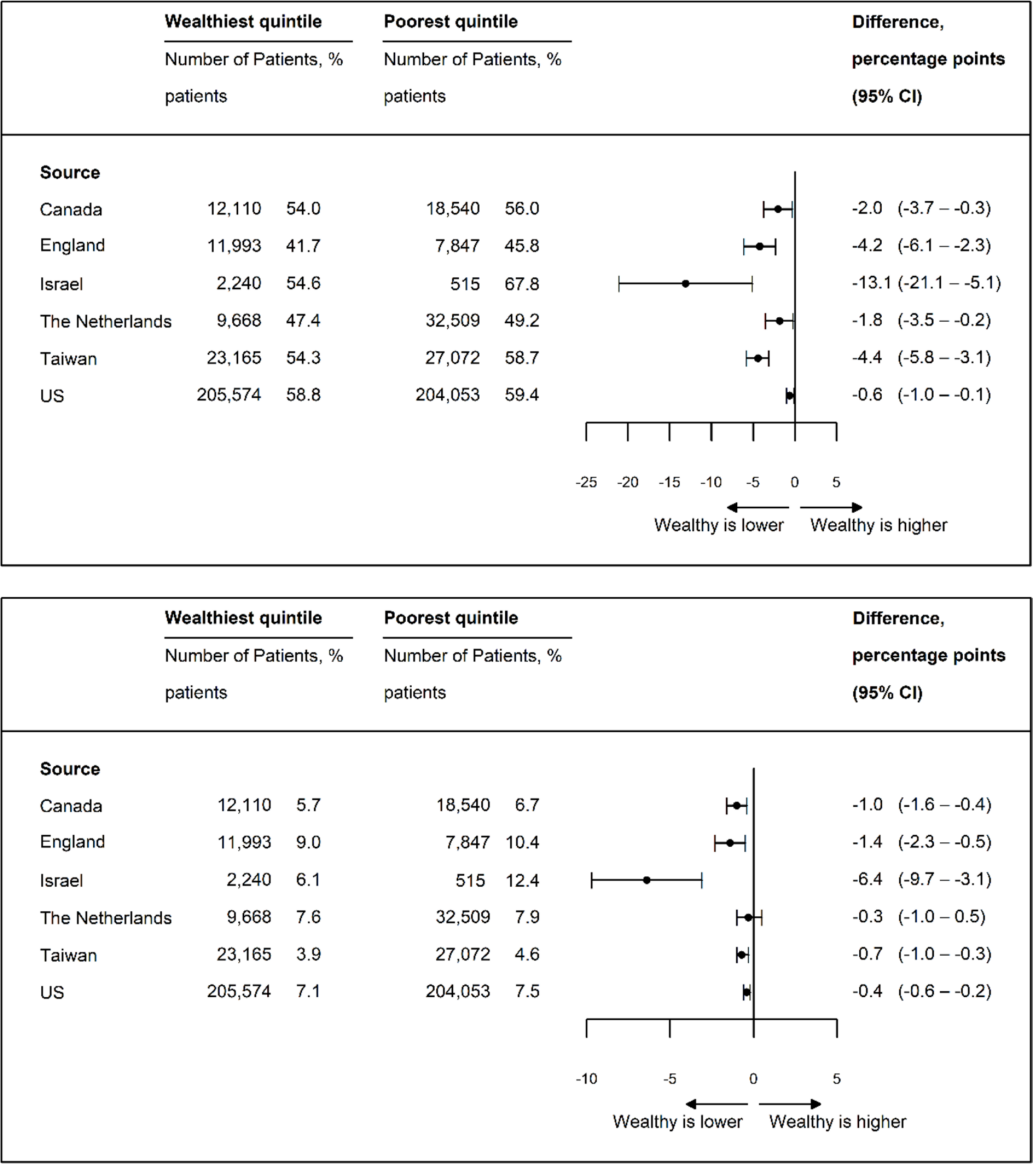
**a** Age- and sex-standardized total hip arthroplasty rate. **b** Age- and sex-standardized hemi-arthroplasty rate. **c** Age- and sex-standardized internal fixation rate. **d** Age- and sex-standardized non-operative rate.

High-income patients were more likely to undergo intensive surgical procedures such as THA and HA (Fig. 2a and b), with the largest differences in Israel for THA (6.9 pp; 95% CI, 4.6–9.2 pp) and for HA (12.6 pp; 95% CI, 8.1–17.0 pp). The high- versus low-income differences in the USA were smaller and resembled those of the other countries: 1.4 pp (95% CI, 1.3–1.5 pp) for THA and 0.4 pp (95% CI, − 0.7 to − 0.1 pp) for HA.

### LOS and Readmission

**Figure 3:**
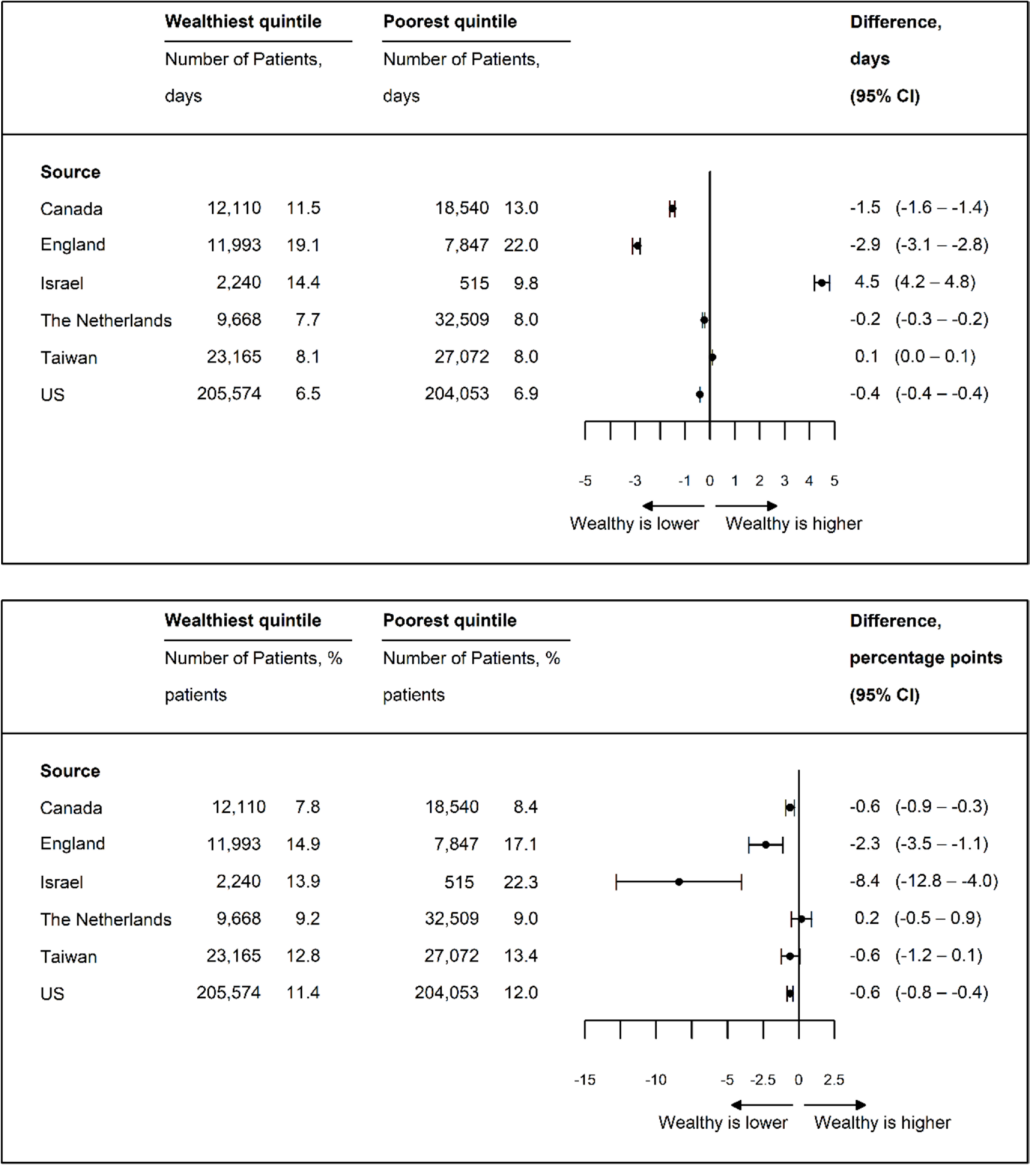
**a** Age- and sex-standardized length of stay (days). **b** Adjusted 30-day readmission rate*. Adjusted for age, sex, and comorbidity.

With the exception of Israel and Taiwan, LOS was shorter for high- versus low-income patients (Fig. 3a). Larger income-based differences in LOS were seen in Canda, England, and Israel. Adjusted 30-day readmission rates were lower for high-income patients except for the Netherlands (Fig. 3b). The differences for high- vs. low-income patients in the other five countries ranged from − 0.6 pp in Canada (95% CI, − 0.9 to − 0.3 pp) to − 8.4 pp in Israel (95% CI, − 12.8 to − 4.0 pp).

### Days from Admission to Surgery and Discharge Disposition

**Figure 4:**
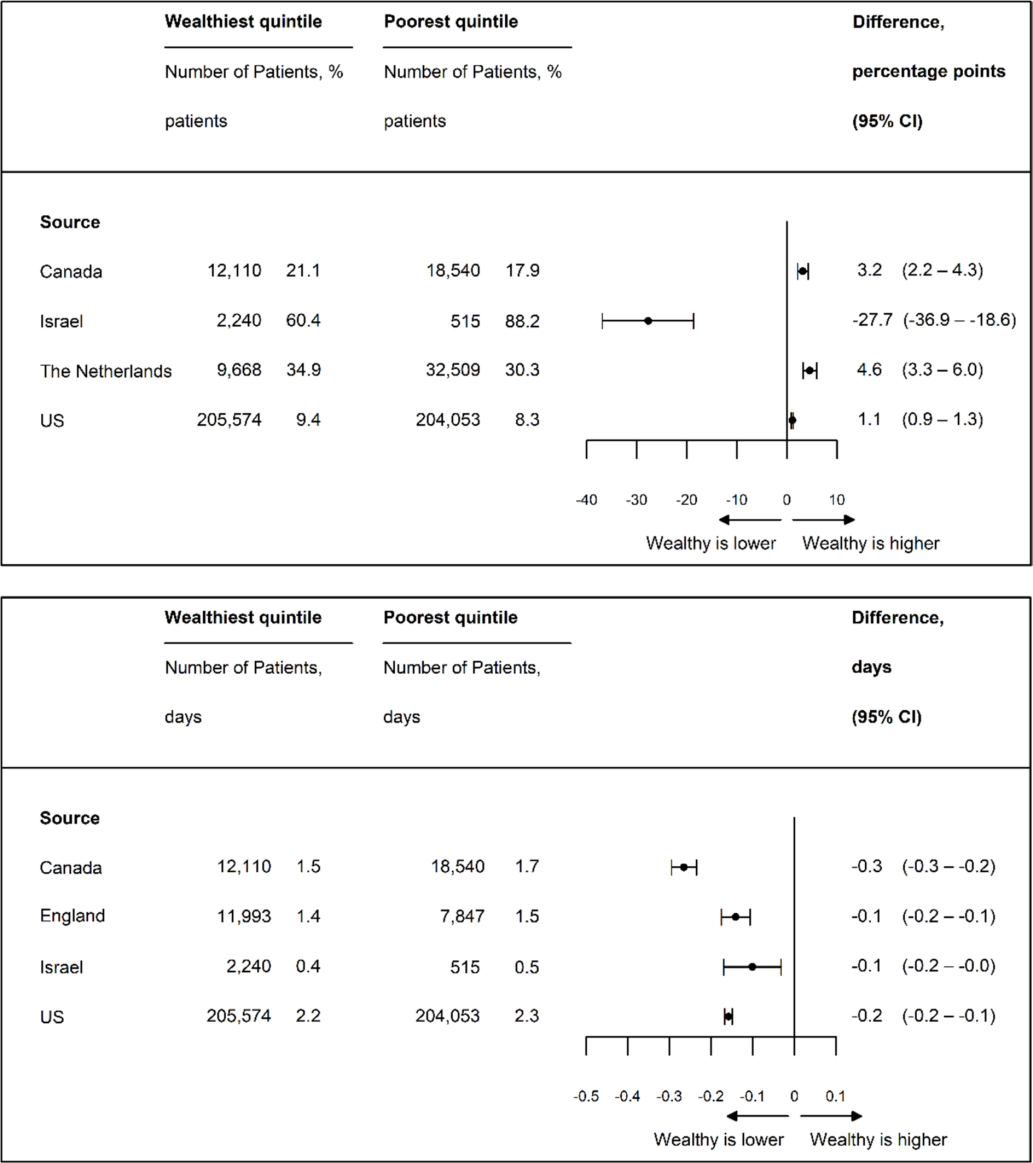
**a** Age- and sex-standardized days from presentation to operation. **b** Age- and sex-standardized percentage of patients discharged to home.

Time from hospital admission to surgery was consistently shorter for high-income patients (Fig. 4a), but the differences were not clinically significant, ranging from − 0.1 days in Israel (95% CI, − 0.2 to − 0.03 days) to − 0.3 days in Canada (95% CI, − 0.3 to − 0.2 days). High-income patients were more likely to be discharged home in the USA, Canada, and the Netherlands, but less likely in Israel, where the overall rate of discharge to home was substantially higher (Fig. 4b). The differences varied from 3.2 pp in Canada (95% CI, 2.2–4.3 pp) to −27.7 pp (95% CI, −36.9–−18.6 pp) in Israel.

## DISCUSSION

In this analysis of patient-level population-representative administrative data from six high-income countries, we identified important within-country income-based differences in treatment and outcomes of patients hospitalized with hip fracture. Across the countries studied, high-income patients were more likely to receive more complex (and costly) surgical repairs, less likely to be readmitted, and less likely to die within 1 year. We also observed difference across countries in the magnitude of the gaps between low- and high-income patients’ LOS and discharge to home. Altogether, the results suggest that for hip fracture, lower-income patients were subject to disparities in treatment process and outcomes in all six countries despite universal health insurance, but the magnitude varied modestly across countries.

As described above, higher mortality observed in low-income patients relative to high-income patients in all six countries may be partially explained by their longer time to surgery and less intensive surgical treatment (THA vs. non-operative management) we observed.^20–22,33,34^ Whereas we have no a priori reason to believe that fracture type, complexity, or acuity of patients would differ systematically by income within country, these findings may warrant further investigations of likely financing and organizational factors compromising timely access to needed surgical care among low-income patients. Similarly, readmission rates among low-income patients were generally higher, which could relate to differences in the availability of social supports, access to rehabilitation and post-acute care, adequacy in care coordination, and availability of community resources, which we could not assess in our study.^35–37^

Unlike previous studies,^1–3,13,17,38,39^ we did not find larger income-based disparities in mortality in the USA. One potential explanation for our finding of a similar “poverty penalty” in the USA as compared with other countries may relate to the fact that in the USA adults aged 65 years and above generally are covered by Medicare and those with lower incomes are dually eligible for Medicaid coverage, which contrasts markedly with the lack of universal health coverage for Americans under age 65. Thus, we might have found significantly larger income-based disparities in the USA relative to other countries if we had used all-payer data or included younger populations in our study; unfortunately, the fragmented nature of US insurance for those under the age of 65 limits our ability to obtain nationally representative data on this population in the USA. Taiwan had the smallest income-based differences in mortality, but also the narrowest gap in average income between high- and low-income areas, which suggests that geographic areas in Taiwan are more heterogeneous with respect to income. In contrast, Israel had the largest income-based differences in mortality rates and the largest gap in average income between patients at the top and bottom quintiles. The findings in Israel reinforce the potential role of income inequality in health disparities.

Finally, our finding that low-income patients were less likely to be discharged home than their high-income peers in Canada, England, and the USA, but substantially more likely to be discharged home than high-income patients in Israel, is interesting. Low-income patients might lack family caregivers and home environment to allow for discharge home while high-income patients could have the support and resources to manage convalescence at home, which likely drives these observed differences. In contrast, in Israel, the vast majority of patients are discharged to home with community-based rehabilitation compared to other countries, which may be attributable to its greater availability of at-home, postoperative services or expectation and preferences of patients, families, and providers. Use of post-acute care in Israel therefore might be more a sign of wealth or privelege.^40^

Our analyses are subject to limitations. First, administrative claims data lack detailed clinical information such as fracture subtype, anatomy, and complexity, which may influence treatment decisions and outcomes. However, we used population-representative data with standardized inclusion and exclusion criteria and coding algorithms to ensure comparable patients across and within countries. Thus, it is unlikely that either the within-country or between-country differences in hip fracture treatment or outcomes can be attributed to differences in patient presentation. Rather, the findings are more likely related to differences in how hip fracture care is organized and funded in each country as well as, potentially, patient, caregiver, and provider preferences. Second, there is no one best method to identify high- versus low-income populations across countries. In this study, we used area-level household income, which is a preferred approach in elderly populations because measures of individual-level income do not account for assets or consumption. Nonetheless, this approach may lead to misclassification, which also varies by the degree of income mixing within areas in each country. Misclassification likely biases our results toward the null. Third, we lacked data on important confounders such as unmeasured comorbidities, pre-fracture functional status, availability of social supports, level of long-term care resources, and patient preferences. The observed income variations in treatments and outcomes across countries may be explained by differences in these demand and supply factors. Further investigation into demand and supply side factors may help in this regard. Fourth, although we could not assess functional outcomes, more intensive surgical approaches along with greater availabililty of social support and long-term care resources may yield better functional outcomes for high-income patients.^20,21,34,41^ Fifth, we were unable to analyze discharge disposition in detail. Future research with more standardized algorithms in categorizing disposition type and more comprehensive data can help to contribute in this regard. Finally, our analyses excluded hip fracture patients aged 65 years and younger, and the Medicare Advantage enrollees in the USA. Thus, these results may not be generalizable to these populations. Furthermore, we also recognize that while focusing our analysis on patients hospitalized with a primary diagnosis of hip fracture is well-established, there is a possibility that a subset of patients with acute hip fractures may be missed, particularly if such patients were given a primary diagnosis related to a complication (e.g., myocardial infarction). However, we applied these criteria uniformly so would not expect this to bias our main results.

## CONCLUSION

In this study of older adults hospitalized with hip fracture in six high-income countries, we found that income-based disparities in treatment and outcomes exist across all countries, with no clear evidence of larger disparities in the USA. Alternatively, we found variation in the magnitude of the income-based differences across countries suggesting that wealth-based disparities may be attenuated by healthcare system design and funding models. Policy makers should consider further efforts to improve accessibility and efficiency of inpatient and post-acute care, and continuity of care among lower-income individuals while orthopedic surgeons and other clinicians should recognize that lower-income patients experiencing hip fracture are at increased risk for adverse outcomes and continue to advocate for systemic changes in healthcare delivery.

## Supplementary Information

Below is the link to the electronic supplementary material.

Supplementary file1 (DOCX 100 KB)

## Data Availability

Datasets are unavailable for sharing because of privacy policies and regulations of participant countries. Our protocol might be available by request from the corresponding author, depending upon the regulatory environment of IHSRC participant countries.
