# Supplementary material for: Comparison of Management and Outcomes of Hip Fractures Among Low- and High-Income Patients in Six High-Income Countries
Source: J Gen Intern Med. 2024 Dec 20;40(11):2602–12. doi: 10.1007/s11606-024-09274-9 (PMC12405133; doi:10.1007/s11606-024-09274-9)
Supplement: Supplementary file 1 — Supplementary file1 (DOCX 100 KB) [file 11606_2024_9274_MOESM1_ESM.docx]

**Appendix Content**

**Appendix eTable I: Description of Data Sources from Each Country/Jurisdiction.**

**Appendix eTable II: List of Codes Used by Each Country to Identify Hip Fractures, Total Hip Arthroplasty (THA), Hemiarthroplasty, and Internal Fixation (IF).**

**Appendix eTable III: Cohorts Generation Steps.**

**Appendix eTable IV: Jurisdiction-Specific Measures of Socioeconomic Status.**

**Appendix eTable V: Concentration of Comorbidities within Study Population.**

**Appendix eFigure 1: Age-Sex Standardized Mortality.**

**Appendix eFigure 2: Age-Sex Standardized 30-Day Readmissions.**

**Statistical Appendix: Calculation of Age-Sex Standardized Estimates**

# Appendix - Variation in Utilization and Outcomes for Patients with Hip Fracture in 6 High-Income Countries: Cross-Sectional Cohort Study

**eTable I: Description of Data Sources from Each Country/Jurisdiction**

**Ontario (Canada)**

All hospitalizations for hip fracture in adults aged> 66 years at the time of hospitalization during calendar years 2013-2019 were identified using 100% Ontario Discharge Abstract Database (DAD). Data from 2012 was used as a “look-back” and data from 2020 were used to ascertain post-hip fracture outcomes.

The DAD includes all admissions to all acute care hospitals in the province of Ontario and thus is inclusive of the entire Ontario population.

| **Hip Fracture Identification and Outcomes** | |
| --- | --- |
| Data source[[1]](#footnote-1) | Use |
| Ontario Discharge Abstract Database (2012-2020) | a. Identification of patients hospitalized with a primary diagnosis of a hip fracture  b. Creation of comorbidities  c. In-hospital outcomes |
| OHIP Billing Data (2013-2019) | a. Post-hip fracture treatments and procedures |
| Registered Persons Data Base files | a. Determination of death date, birth date, and insurance coverage start and end date |
| **Population Count and Demographics** (Used for Calculation of hip fracture Hospitalization Rates and Standardization) | |
| Registered Persons Data Base files | Age/sex/ethnicity/area of residence information |

**Manitoba (Canada)**

All hospitalizations for hip fractures in adults aged> 66 years at the time of hospitalization during calendar years 2013-2019 were identified using 100% Manitoba Discharge Abstract Database (DAD). Data from 2012 was used as a “look-back” and data from 2020 were used to ascertain post-hip fracture outcomes.

The Manitoba DAD includes all admissions to all acute care hospitals in the province of Manitoba and thus is inclusive of the entire Manitoba population eligible to receive health services.

Analyses were conducted using the Population Research Data Repository housed at the Manitoba Centre for Health Policy at the University of Manitoba and utilized the administrative data from the Manitoba Ministry of Health (I.e., Manitoba Health and Seniors Care). The administrative data captures all publicly-insured health services for all residents of the province who are eligible to receive health services.

| **Hip Fracture Identification and Outcomes** | |
| --- | --- |
| Data source[[2]](#footnote-2) | Use |
| Manitoba Discharge Abstract Database (2012-2020) | a. Identification of patients hospitalized with a primary diagnosis of a hip fracture  b. Creation of comorbidities  c. In-hospital outcomes |
| Physician Billing Data (2013-2019) | a. Post-hip fracture treatments and procedures |
| Manitoba Health Insurance Registry | Determination of death date, birth date, and insurance coverage start and end dates. |
| **Population Count and Demographics** (Used for Calculation of hip fracture Hospitalization Rates and Standardization) | |
| Manitoba Health Population Database | Counts of the population by sex, age group, and year |

**England**

All hospitalizations for hip fracture in adults aged> 66 years at the time of hospitalization during calendar years 2013-2018 were identified using the Clinical Practice Research Datalink. Data from 2012 was used as a “look-back” and data from 2019 were used to ascertain post-hip fracture outcomes. CPRD has been shown to be representative of the English population by age, sex, and ethnicity and has been validated for research.^1-3^ Analyses were conducted at the Institute of Health Informatics, University College London using linked electronic health records from primary care (CPRD), hospitalization (HES), and the national death registry (ONS).^2^

| **Hip Fracture Identification and Outcomes** | |
| --- | --- |
| Data source | Use |
| Clinical Practice Research Datalink (GOLD and Aurum) | a. Patient identification  b. Demographics  c. Creation of comorbidities |
| Hospital Episode Statistics (HES) | a. Identification of patients hospitalized with a primary diagnosis of hip fracture  b. Creation of comorbidities  c. In-hospital outcomes  d. Inpatient procedures |
| Office of National Statistics | Cause-specific mortality |
| **Population Count and Demographics** (Used for Calculation of hip fracture Hospitalization Rates and Standardization) | |
|  | Counts of population by sex, age group, and year |

**Israel**

Analyses were conducted at the Clinical Research Center (CRC) in Soroka University Medical Center and included national data of Clalit Health Services insured patients.

All hospitalizations for hip fracture in adults aged> 66 years at the time of hospitalization during calendar years 2013-2019 were identified using Clalit Health Services (CHS) Data sharing platform powered by MDClone© (<https://www.mdclone.com>). Data from 2012 was used as a “look-back” and data from 2020 were used to ascertain post-hip fracture outcomes. The data is broadly representative of the Israel population concerning age, sex, and geography. Clalit Health Services is Israel’s largest insurance company and healthcare provider, providing most of Israel’s healthcare services and health insurance to 54% of the country’s population. Services include primary, secondary, and tertiary care (including a third of Israel’s acute care beds), pharmacies, and paramedical services. CHS maintains a comprehensive database, continuously updated with information about a subject’s demographics, community and outpatient visits, laboratory tests, hospitalizations, medication prescriptions, and purchases.

| **Hip Fracture Identification and Outcomes** | |
| --- | --- |
| Data sources[[3]](#footnote-3) | Use |
| CHS Data warehouse using accessed by MDClone© | a. Identification of patients hospitalized with a primary diagnosis of a hip fracture  b. Demographic information (Age, sex, SES)  c. Creation of comorbidities |
| CHS Data warehouse using accessed by MDClone© | d. Procedures related to the diagnosis of hip fracture  e. In-hospital and post-hospital utilization and outcomes |
| **Population Count and Demographics** (Used for Calculation of hip fracture Hospitalization Rates) | |
| CHS Data warehouse using accessed by MDClone© | The population denominator includes all Clalit members that were > 66 years in each year from 2013 through 2019 and were members of Clalit one year before and after that year. |

**Netherlands**

All hospitalizations for hip fracture in Dutch adults aged> 66 years at the time of hospitalization during calendar years 2013-2019 were identified using data from the national register for hospital care.^4^ Data from 2012 was used as a “look-back” and data from 2020 were used to ascertain post-hip fracture outcomes.

These data include all inpatient hospitalizations in the Netherlands. Primary and secondary diagnoses are recorded, as well as the main procedure performed during the admission. Demographic information and, if applicable, the date of death were extracted from municipality registers[[4]](#footnote-4).

Due to a certain number of patients each year lacking registration of their operations, we took the precaution of upscaling the surgery performed for the remaining patients to reach 100%. This was done to prevent any unjustified exclusions. To ensure that this approach did not introduce any bias, we conducted a sensitivity analysis. The results showed that the patients with missing registrations were equally distributed across geographical regions and hospitals.

| **Hip Fracture Identification and Outcomes** | |
| --- | --- |
| Data sources[[5]](#footnote-5) | Use |
| National Register for hospital care, Landelijke Basisregistratie Ziekenhuiszorg (LBZ – 2013-2020), and Landelijke Medische Registratie (LMR – 2012) | a. Identification of patients hospitalized with a primary diagnosis of a hip fracture  b. Creation of comorbidities  c. In-hospital and post-hospital procedure utilization[[6]](#footnote-6) and outcomes |
| Municipality Register (GBAPERSOONTAB, GBAOVERLIJDENTAB) | Determination of age and sex, date of death |
| **Population Count and Demographics** (Used for Calculation of hip fracture Hospitalization Rates and Standardization) | |
| Municipality Register (GBA) | Counts of population by sex, age group, and year |

**Taiwan**

All hospitalizations for hip fracture in adults aged> 66 years at the time of hospitalization during calendar years 2013-2019 were identified using Taiwan’s Inpatient Expenditures data. Data from 2012 was used as a “look-back” and data from 2020 were used to ascertain post-hip fracture outcomes.

Key data files included the National Health Insurance (NHI) Inpatient Expenditures by Admission file, which contains all admissions to all acute care hospitals in Taiwan and thus is inclusive of Taiwan population. The NHI Registry for Beneficiaries file includes demographic and socioeconomic status information of all NHI beneficiaries. Because all legal residents in Taiwan are eligible to enroll in the NHI program and the enrollment rate has exceeded 99% since 2000, these data are population representative. The Cause of Death Data provides information on the cause and date of all reported deaths in Taiwan. Analyses were conducted at the Yang-Ming branch of the Health and Welfare Data Science Center, Taiwan Ministry of Health and Welfare. The primary data sources were the National Health Insurance Research Database and Cause of Death Data.^5^

| **Hip Fracture Identification and Outcomes** | |
| --- | --- |
| Data sources[[7]](#footnote-7) | Use |
| Inpatient Expenditures by Admissions (2012-2020) | a. Identification of patients hospitalized with a primary diagnosis of a hip fracture^6^  b. Creation of comorbidities  c. In-hospital and post-hospital utilization and outcomes |
| Cause of Death Data | Determination of death date |
| **Population Count and Demographics** (Used for Calculation of hip fracture Hospitalization Rates and Standardization) | |
| Registry for Beneficiaries | Counts of the population by sex, age group, and year  Determination of birth date and sex |

**United States**

All hospitalizations for hip fracture in adults aged> 66 years at the time of hospitalization during calendar years 2013-2019 were identified using 100% Medicare fee-for-service (FFS) data. Data from 2012 was used as a “look-back” and data from 2020 were used to ascertain post-hip fracture outcomes.

During our study period, approximately 75% (2011) and 67% (2018) of the US Medicare population were enrolled in FFS Medicare and thus included in our study population, with the remainder enrolled in Medicare managed care plans.

| **Hip Fracture Identification and Outcomes** | |
| --- | --- |
| Data sources[[8]](#footnote-8) | Use |
| 100% Medicare Part A Data (2012-2020) | a. Identification of patients hospitalized with a primary diagnosis of hip fracture  b. Creation of comorbidities  c. In-hospital and post-hospital utilization and outcomes |
| 100% Medicare Beneficiary Summary File | Date of death |
| **Population Count and Demographics** (Used for Calculation of hip fracture Hospitalization Rates and Standardization) | |
| Medicare Beneficiary Summary File | Number of Medicare FFS enrollees by year  Age/race/sex/ethnicity/area of residence information |

Analyses were conducted at Harvard Medical School.

**References**

1. Herrett E, Shah AD, Boggon R, et al. Completeness and diagnostic validity of recording acute myocardial infarction events in primary care, hospital care, disease registry, and national mortality records: cohort study. *Bmj* 2013;346:f2350. doi: 10.1136/bmj.f2350 [published Online First: 2013/05/23]

2. Herrett E, Gallagher AM, Bhaskaran K, et al. Data Resource Profile: Clinical Practice Research Datalink (CPRD). *Int J Epidemiol* 2015;44(3):827-36. doi: 10.1093/ije/dyv098 [published Online First: 2015/06/08]

3. Wolf A, Dedman D, Campbell J, et al. Data resource profile: Clinical Practice Research Datalink (CPRD) Aurum. Int J Epidemiol. Dec 1 2019;48(6):1740-1740g. doi:10.1093/ije/dyz034

4. World Health Organization. Regional Office for E, European Observatory on Health S, Policies, et al. Netherlands: health system review. Copenhagen: World Health Organization. Regional Office for Europe 2016:240 p.

5. Hsieh CY, Su CC, Shao SC, et al. Taiwan’s National Health Insurance Research Database: past and future. *Clin Epidemiol* 2019;11:349-58. doi: 10.2147/clep.S196293 [published Online First: 2019/05/24]

6. Saw SM, Hong CY, Lee J, et al. Awareness and health beliefs of women towards osteoporosis. *Osteoporos Int* 2003;14(7):595-601.

**eTable II: List of Codes Used by Each Country to Identify Hip Fractures, Total Hip Arthroplasty (THA), Hemiarthroplasty (HA), and Internal Fixation (IF)[[9]](#footnote-9)**

ICD = International Classification of Diseases, CCI = Canadian Classification of Interventions.

*** Patients who had multiple procedures during the index admission were assigned to the most intensive type of repair (THA>HA>IF).**

|  | Canada | England | Israel | Netherlands | Taiwan | United States |
| --- | --- | --- | --- | --- | --- | --- |
| Hip fracture diagnosis in admission | ICD-10-CA:  S72.0x  S72.1x  S72.2x | ICD-10:  S72.0x  S72.1x  S72.2x | ICD-9:  820.0x  820.20  820.21  820.1  820.22  820.3  820.8  820.9 | ICD-10:  S72.0x  S72.1x  S72.2x | ICD-9:  820.0x  820.20  820.21  820.1  820.22  820.3  820.8  820.9  Icd-10:  S72.0x  S72.1x  S72.2x | ICD-9:  820.0x  820.20  820.21  820.1  820.22  820.3  820.8  820.9  Icd-10:  S72.0x  S72.1x  S72.2x |
| Codes for THA, HA, and IF by country | | | | | | |
| THA | CCI:  1.VA.53.LA.PN | OPCS-4:  W37.1  W37.8  W37.9  W38.1  W38.8  W38.9  W39.1  W39.8  W39.9  W93.1  W93.8  W93.9  W94.1  W94.8  W94.9  W95.1  W95.8  W95.9 | 81.51 | ZA codes:  038567  190305  038569  038568  CVV codes:  58150  581652  581520  CBV codes:  338568K  338568J  338568A  338567K  338568B  338567J  338568L  338568P  338569K  338568W  338569D  338568U  338567A  338568Z  338569 | ICD-9:  81.51  ICD-10:  0SRB019  0SRB0J9  0SRB0JA  0SRB0JZ   0SRB01A   0SRB01Z   0SRB029   0SRB02A   0SRB02Z   0SRB039   0SRB03A  0SRB03Z   0SRB049   0SRB04A   0SRB04Z  0SRB069  0SRB06A  0SRB06Z  0SRB07Z  0SRB0EZ  0SRB0J9  0SRB0JA  0SRB0JZ  0SRB0KZ  Right= 9  0SR90JA  0SR90JZ  0SR9019  0SR901A  0SR901Z  0SR902A  0SR902Z  0SR9039  0SR903A  0SR903Z  0SR9049  0SR904A  0SR904Z  0SR9069  0SR906A  0SR906Z  0SR907Z  0SR90EZ  0SR90J9  0SR90JA  0SR90JZ  0SR90KZ  0SR9019  0SR9029 | ICD-9:  81.51  ICD-10:  0SRB019  0SRB0J9  0SRB0JA  0SRB0JZ   0SRB01A   0SRB01Z   0SRB029   0SRB02A   0SRB02Z   0SRB039   0SRB03A  0SRB03Z   0SRB049   0SRB04A   0SRB04Z  0SRB069  0SRB06A  0SRB06Z  0SRB07Z  0SRB0EZ  0SRB0J9  0SRB0JA  0SRB0JZ  0SRB0KZ  Right= 9  0SR90JA  0SR90JZ  0SR9019  0SR901A  0SR901Z  0SR902A  0SR902Z  0SR9039  0SR903A  0SR903Z  0SR9049  0SR904A  0SR904Z  0SR9069  0SR906A  0SR906Z  0SR907Z  0SR90EZ  0SR90J9  0SR90JA  0SR90JZ  0SR90KZ  0SR9019  0SR9029 |
| HA | CCI:  1.VA.53.LA.PM, 1.SQ.53.LA.PM | OPCS-4:  W46.1  W46.8  W46.9  W47.1  W47.8  W47.9  W48.1  W48.8  W48.9 | 81.52 | ZA codes:  038565  190376  190344  038570  038524  038560  CVV codes:  58160  581651  581521  581513  581525  581522  581523  CBV codes:  338567  338567H  338567W  338567L  338567R  338567D  338567E  190344  190345  338567F  338567G  338567C  338567Y | Icd-9:  81.52  Icd-10:  0SR.A009  0SR.A00A  0SR.A00Z  0SR.A019  0SR.A01A  0SR.A01Z  0SR.A039  0SR.A03A  0SR.A03Z  0SR.A07Z  0SR.A0J9  0SR.A0JA  0SR.A0JZ  0SR.S019  0SR.S01A  0SR.S01Z  0SR.S039  0SR.S03A  0SR.S03Z  0SR.S07Z  0SR.S0J9  0SR.S0JA  0SR.S0JZ  0SR.S0KZ  Right= E, R  0SR.E009  0SR.E00A  0SR.E00Z  0SR.E019  0SR.E01A  0SR.E01Z  0SR.E039  0SR.E03A  0SR.E03Z  0SR.E07Z  0SR.E0J9  0SR.E0JA  0SR.E0JZ  0SR.E0KZ  0SR.R019  0SR.R01A  0SR.R01Z  0SR.R039  0SR.R03A  0SR.R03Z  0SR.R07Z  0SR.R0J9  0SR.R0JZ  0SR.R0KZ | Icd-9:  81.52  Icd-10:  0SR.A009  0SR.A00A  0SR.A00Z  0SR.A019  0SR.A01A  0SR.A01Z  0SR.A039  0SR.A03A  0SR.A03Z  0SR.A07Z  0SR.A0J9  0SR.A0JA  0SR.A0JZ  0SR.S019  0SR.S01A  0SR.S01Z  0SR.S039  0SR.S03A  0SR.S03Z  0SR.S07Z  0SR.S0J9  0SR.S0JA  0SR.S0JZ  0SR.S0KZ  Right= E, R  0SR.E009  0SR.E00A  0SR.E00Z  0SR.E019  0SR.E01A  0SR.E01Z  0SR.E039  0SR.E03A  0SR.E03Z  0SR.E07Z  0SR.E0J9  0SR.E0JA  0SR.E0JZ  0SR.E0KZ  0SR.R019  0SR.R01A  0SR.R01Z  0SR.R039  0SR.R03A  0SR.R03Z  0SR.R07Z  0SR.R0J9  0SR.R0JZ  0SR.R0KZ |
| Internal fixation | CCI:  1.VA.74, 1.VC.74 | OPCS-4:  W19.1 P  W24.1 | 79.35  78.55  79.25  79.15 | ZA codes:  038535  038533  038534  038556  038527  038532  038529  038426  038525  CVV codes:  579013  579096  579267  579056  579266  579260  579269  579264  579223  579216  579263  579268  579262  579006  579261  579296  82043  578706  579024  579086  579265  82049  578726  CBV codes:  338534R  338534E  338536X  338533Y  338533Q  338534S  338533  338534P  338536  338533X  338534G  338534Q  338539L  338533W  338534N  338533R  338536F  338534R  338533G  338533H  338536E  338533C  338534J  338536B  338533D  38556  338534  338535N  338539K  338535R  338536R  338535S  038532  338534S  338534T  038512  338536J  338533U  338536N  338534L  338533M  338534I  338537J  338538E  338538F  338538N  038525  338527  338533B  338533F  338533T  338534K  338534M  338536W  338537Y  338539H | Icd-9:  79.35  78.55  79.25  79.15  Icd-10:  0QS.604Z  0QS.704Z  0QS.804Z  0QS.904Z  0QS.B04Z  0QS.C04Z  0QH.604Z  0QH.634Z  0QH.644Z  0QH.704Z  0QH.734Z  0QH.744Z  0QH.804Z  0QH.834Z  0QH.844Z  0QH.904Z  0QH.934Z  0QH.944Z  0QH.B04Z  0QH.B34Z  0QH.B44Z  0QH.C04Z  0QH.C34Z  0QH.C44Z  0QS.60ZZ  0QS.70ZZ  0QS.80ZZ  0QS.90ZZ  0QS.B0ZZ  0QS.C0ZZ  0QS706Z  0QS606Z  0QH706Z  0QH606Z  0QS906Z  0QS806Z  0QS806Z  0QSB06Z  0SSB04Z  0SS904Z  0QSC06Z  0QH906Z  0QH806Z  0QHB06Z  0QHC06Z  0SHB04Z  0SHB04Z  0SH904Z  0QS.634Z  0QS.644Z  0QS.734Z  0QS.744Z  0QS.834Z  0QS.844Z  0QS.934Z  0QS.944Z  0QS.B34Z  0QS.B44Z  0QS.C34Z  0QS.C44Z  0QS736Z  0QS636Z  0SRR0JA  0QH736Z  0QH636Z  0QS6XZZ  0QS7XZZ  0QSB36Z  0QS646Z  0QS836Z  0QS936Z  0QS746Z  0SSB34Z  0SS934Z  0QSC36Z  0QH936Z  0QH836Z  0QHB36Z  0QS635Z  0QS735Z  0QHC36Z  0SHB34Z  0QH746Z  0SH934Z | Icd-9:  79.35  78.55  79.25  79.15  Icd-10:  0QS.604Z  0QS.704Z  0QS.804Z  0QS.904Z  0QS.B04Z  0QS.C04Z  0QH.604Z  0QH.634Z  0QH.644Z  0QH.704Z  0QH.734Z  0QH.744Z  0QH.804Z  0QH.834Z  0QH.844Z  0QH.904Z  0QH.934Z  0QH.944Z  0QH.B04Z  0QH.B34Z  0QH.B44Z  0QH.C04Z  0QH.C34Z  0QH.C44Z  0QS.60ZZ  0QS.70ZZ  0QS.80ZZ  0QS.90ZZ  0QS.B0ZZ  0QS.C0ZZ  0QS706Z  0QS606Z  0QH706Z  0QH606Z  0QS906Z  0QS806Z  0QS806Z  0QSB06Z  0SSB04Z  0SS904Z  0QSC06Z  0QH906Z  0QH806Z  0QHB06Z  0QHC06Z  0SHB04Z  0SHB04Z  0SH904Z  0QS.634Z  0QS.644Z  0QS.734Z  0QS.744Z  0QS.834Z  0QS.844Z  0QS.934Z  0QS.944Z  0QS.B34Z  0QS.B44Z  0QS.C34Z  0QS.C44Z  0QS736Z  0QS636Z  0SRR0JA  0QH736Z  0QH636Z  0QS6XZZ  0QS7XZZ  0QSB36Z  0QS646Z  0QS836Z  0QS936Z  0QS746Z  0SSB34Z  0SS934Z  0QSC36Z  0QH936Z  0QH836Z  0QHB36Z  0QS635Z  0QS735Z  0QHC36Z  0SHB34Z  0QH746Z  0SH934Z |

**eTable III: Cohorts Generation Steps (Please update the numbers to 2019)**

| Country | Canada | England** | Israel | Netherlands | Taiwan | US* |
| --- | --- | --- | --- | --- | --- | --- |
| Initial Cohort | 91,471 | 75,982 | 28,281 | 139,344 | 168,293 | 1,868,183 |
| Step 1: Exclude if not first hip fracture admission in the past 180 days (look back 180 days from the index date to the prior DISCHARGE date) | 1,430 | 10,020 | 1,063 | 2,993 | 7,016 | 116,196 |
| Step 2: Delete patients with missing age or sex | 10,445 | 0 | 0 | 0 | 712 | 69 |
| Step 3: Delete Age<66 |  | 6,318 | 3,398 | 19,430 | 30,950 | 114,642 |
| Step 4: Delete Episodes Associated with Trauma^***^ | 4,400 | 3,960 | 1,670 | 15,363 | 9,479 | 3732 |
| Step 5: Resides out of area | 452 | 0 | 2 | 72 | 0 | 512,946 |
| Step 6: Exclude if without 12-month continuous enrolment (pre-index) |  | 1,494 | 182 | 0 | 0 | 39169 |
| Step 7: Exclude if without 12-month continuous enrolment unless died within one year (post index) | 96 | 3,789 | 19 | 0 | 0 | 458 |
| Step 8: Exclude if 2+ months consecutive MA within 12 months prior to the index admission | NA | NA | NA | NA | NA | 152,25 |
| Step 9: Exclude if 2+ months consecutive MA within 12 months post-index admission | NA | NA | NA | NA | NA | 19,892 |
| Step 10: Exclude if without information regarding SES | 1,136 | 46 | 1,142 | 4,763 | NA | NA |
| Final cohort | 73,512 | 50,355 | 20,805 | 96,723 | 120,136 | 1,045,854 |

* Because complete data on in-hospital and out-of-hospital treatments was lacking for patients enrolled in Medicare Advantage (MA) plans, we excluded them from the analysis.

**England reflects cases from 2013 to 2018.

***The following ICD-10 codes or equivalent were used to exclude hip fractures associated with trauma: E800-E848, E881-884, E908-909, E916-928^***^

**eTable IV: Jurisdiction-Specific Measures of Socioeconomic Status**

| Jurisdiction | SES measure | Unit of measurement | Unit of stratification |
| --- | --- | --- | --- |
| Canada(Manitoba) | Manitoba urban and rural income quintiles | Dissemination area (DA) | Municipal code (or residence) within MB cities with population ≥ 50,000 (urban) vs. outside of cities (rural) |
| Canada(Ontario) | Census Canada income quintiles | Dissemination area | A census metropolitan area (CMA) or a census agglomeration (CA) |
| England | Index of Multiple Deprivation (IMD) | Lower layer super output area (LSOA)Postal code | None |
| Israel | Socioeconomic score 1-5 (SES) measurement including Income, demographic composition, education, standard of living, employment, and retirement. | Neighborhood (statistical areas) | None (sensitivity analysis stratified by North, Central, South) |
| Netherlands | Income | Household | NUTS2 (nomenclature of territorial units for statistics) |
| Taiwan | Median neighborhood income | Township | NHI regional divisions |
| USA | Median household income | ZCTA | Hospital referral region |

**eTable V: Concentration of Comorbidities within Study Population, %.**

|  | **Canada** | | **England** | | **Israel** | | **Netherlands** | | **Taiwan** | | US* | |
| --- | --- | --- | --- | --- | --- | --- | --- | --- | --- | --- | --- | --- |
| **Condition** | 1^st^ (poorest) quintile | 5^th^ (wealthiest) quintile | 1^st^ (poorest) quintile | 5^th^ (wealthiest) quintile | 1^st^ (poorest) quintile | 5^th^ (wealthiest) quintile | 1^st^ (poorest) quintile | 5^th^ (wealthiest) quintile | 1^st^ (poorest) quintile | 5^th^ (wealthiest) quintile | 1^st^ (poorest) quintile | 5^th^ (wealthiest) quintile |
| Congestive Heart Failure | 0.05 | 0.04 | 0.1452 | 0.1194 | 0.16 | 0.12 | 0.009 | 0.007 | 0.07 | 0.06 | 0.23 | 0.21 |
| Cardiac Arrhythmia | 0.06 | 0.06 | 0.3081 | 0.2997 | 0.18 | 0.25 | 0.184 | 0.163 | 0.06 | 0.07 | 0.37 | 0.39 |
| Valvular Disease | 0.01 | 0.01 | 0.1050 | 0.0984 | 0.07 | 0.09 | 0.074 | 0.065 | 0.05 | 0.04 | 0.14 | 0.16 |
| Pulmonary Circulation Disorders | 0.01 | 0.00 | 0.0115 | 0.0090 | 0.03 | 0.03 | 0.015 | 0.011 | 0.00 | 0.00 | 0.03 | 0.03 |
| Peripheral Vascular Disorders | 0.02 | 0.02 | 0.0738 | 0.0450 | 0.05 | 0.07 | 0.051 | 0.041 | 0.01 | 0.01 | 0.11 | 0.10 |
| Hypertension Uncomplicated | 0.29 | 0.26 | 0.6002 | 0.5883 | 0.64 | 0.57 | 0.273 | 0.227 | 0.46 | 0.44 | 0.60 | 0.59 |
| Hypertension Complicated | 0.00 | 0.00 | 0.0043 | 0.0028 | 0.06 | 0.06 | 0.004 | 0.004 | 0.15 | 0.17 | 0.27 | 0.25 |
| Paralysis | 0.01 | 0.00 | 0.0257 | 0.0166 | 0.02 | 0.01 | 0.013 | 0.012 | 0.01 | 0.01 | 0.01 | 0.01 |
| Other Neurological Disorders | 0.04 | 0.04 | 0.0965 | 0.0972 | 0.07 | 0.12 | 0.051 | 0.067 | 0.05 | 0.06 | 0.15 | 0.16 |
| Chronic Pulmonary Disease | 0.09 | 0.07 | 0.2902 | 0.1811 | 0.11 | 0.08 | 0.126 | 0.082 | 0.10 | 0.07 | 0.28 | 0.24 |
| Diabetes Uncomplicated | 0.07 | 0.07 | 0.1919 | 0.1428 | 0.47 | 0.23 | 0.180 | 0.113 | 0.27 | 0.26 | 0.19 | 0.15 |
| Diabetes Complicated | 0.13 | 0.09 | 0.0191 | 0.0132 | 0.10 | 0.03 | 0.017 | 0.013 | 0.05 | 0.06 | 0.10 | 0.08 |
| Hypothyroidism | 0.03 | 0.03 | 0.1172 | 0.1164 | 0.07 | 0.16 | 0.025 | 0.021 | 0.00 | 0.01 | 0.24 | 0.27 |
| Renal Failure | 0.04 | 0.04 | 0.1926 | 0.1658 | 0.18 | 0.16 | 0.111 | 0.079 | 0.08 | 0.11 | 0.22 | 0.21 |
| Liver Disease | 0.01 | 0.01 | 0.0205 | 0.0141 | 0.03 | 0.03 | 0.009 | 0.010 | 0.03 | 0.03 | 0.02 | 0.02 |
| Peptic Ulcer Disease excluding bleeding | 0.00 | 0.00 | 0.0120 | 0.0106 | 0.01 | 0.01 | 0.001 | 0.000 | 0.04 | 0.03 | 0.01 | 0.01 |
| AIDS/HIV |  |  | 0.0000 | 0.0000 | 0.00 | 0.00 | 0.000 | 0.000 | 0.00 | 0.00 | 0.00 | 0.00 |
| Lymphoma | 0.00 | 0.01 | 0.0070 | 0.0082 | 0.01 | 0.02 | 0.005 | 0.009 | 0.00 | 0.00 | 0.01 | 0.01 |
| Metastatic Cancer | 0.01 | 0.01 | 0.0246 | 0.0219 | 0.02 | 0.01 | 0.015 | 0.020 | 0.01 | 0.01 | 0.01 | 0.01 |
| Solid Tumor without Metastasis | 0.04 | 0.04 | 0.0686 | 0.0637 | 0.07 | 0.15 | 0.032 | 0.036 | 0.04 | 0.05 | 0.04 | 0.04 |
| Rheumatoid Arthritis/collagen | 0.01 | 0.01 | 0.0610 | 0.0588 | 0.02 | 0.05 | 0.024 | 0.020 | 0.01 | 0.01 | 0.05 | 0.05 |
| Coagulopathy | 0.01 | 0.02 | 0.0117 | 0.0093 | 0.01 | 0.01 | 0.011 | 0.012 | 0.01 | 0.01 | 0.10 | 0.10 |
| Obesity | 0.01 | 0.01 | 0.0206 | 0.0156 | 0.13 | 0.06 | 0.008 | 0.005 | 0.00 | 0.00 | 0.06 | 0.05 |
| Weight Loss | 0.02 | 0.01 | 0.0331 | 0.0206 | 0.03 | 0.06 | 0.008 | 0.006 | 0.00 | 0.00 | 0.12 | 0.10 |
| Fluid and Electrolyte Disorders | 0.10 | 0.09 | 0.2084 | 0.1783 | 0.09 | 0.11 | 0.094 | 0.083 | 0.10 | 0.08 | 0.41 | 0.41 |
| Blood Loss Anemia | 0.00 | 0.00 | 0.0031 | 0.0023 | 0.01 | 0.00 | 0.008 | 0.006 | 0.05 | 0.03 | 0.03 | 0.03 |
| Deficiency Anemia | 0.02 | 0.02 | 0.0686 | 0.0499 | 0.14 | 0.11 | 0.033 | 0.024 | 0.01 | 0.01 | 0.07 | 0.06 |
| Alcohol Abuse | 0.02 | 0.01 | 0.0521 | 0.0267 | 0.00 | 0.00 | 0.016 | 0.022 | 0.00 | 0.00 | 0.03 | 0.02 |
| Drug Abuse | 0.00 | 0.00 | 0.0018 | 0.0008 | 0.01 | 0.00 | 0.001 | 0.000 | 0.00 | 0.00 | 0.01 | 0.01 |
| Psychoses | 0.01 | 0.00 | 0.0131 | 0.0073 | 0.03 | 0.02 | 0.007 | 0.001 | 0.01 | 0.01 | 0.03 | 0.02 |
| Depression | 0.03 | 0.02 | 0.1030 | 0.0818 | 0.07 | 0.18 | 0.007 | 0.006 | 0.01 | 0.02 | 0.19 | 0.20 |

**eFigure 1: Age-Sex Standardized Mortality**

1. **30-day mortality**

1. **1-year mortality**

**eFigure 2: Age-Sex Standardized 30-Day Readmissions**

**Statistical Appendix: Calculation of Age-Sex Standardized Estimates**

Age-sex-standardized hip fracture rates

For the **whole eligible sample** (i.e., anyone who could be included in the hip fracture population based on the other eligibility criteria), we computed hip fracture rates standardized to the age-sex distribution of the bottom 20% of SES of the whole jurisdiction.

1. Assign each person a value of SES (individual- or neighborhood-level measure)
2. For each region
   1. Identify quintiles of SES: bottom 20%, second 20%, third 20%, fourth 20%, top 20%
   2. To each person, assign a variable equal to their regional quintile of SES, $q_{i}\in\left\{ 1,\ldots,5 \right\}$
3. For the whole jurisdiction, compute standardization weights: the age-sex distribution in the bottom 20% of SES
   $w_{a,s}=\frac{N_{a,s,1}}{N_{1}}$ where $N_{a,s,q}$ is the number of people in age group $a$, sex group $s$, and SES quintile $q$ and $N_{q}=\sum_{a,s} N_{a,s,q}$ is the number of people in quintile $q$ (i.e., collapsed over age and sex)
4. Compute the hip fracture rates$:$
   $r_{a,s,q}= \frac{n_{a,s,q}}{N_{a,s,q}}$ where $n_{a,s,q}$ is the number of people with hip fracture in age group $a$, sex group $s$, and SES quintile $q$
5. Multiply hip fracture rates by weights and sum over the age-sex groups:
$\tilde{r}_{q}= \sum_{a,s} w_{a,s}r_{a,s,q}$
6. Report age-sex standardized hip fracture rates in each SES quintile: $\tilde{r}_{q} for q\in\left\{ 1,\ldots5 \right\}$

To form confidence intervals around these rates, we use Gamma approximation of Fay & Feuer 1997

1. Estimate the variance of the age-sex adjusted rate using:

$s_{q}^{2}= \sum_{a,s} w_{a,s}^{2} \frac{r_{a,s,q}}{N_{a,s,q}}$
2. Compute the lower bound of a 95% confidence interval:
   $LB_{q}=\frac{s_{q}^{2}}{{2 \tilde{r}}_{q}}\left( \chi_{2 \tilde{r}_{q}^{2}/s_{q}^{2}}^{2} \right)^{-1}\left( .025 \right)$ where $\left( \chi_{df}^{2} \right)^{-1}\left( \alpha\right)$ is the $100\alpha$ %ile of a Chi-square distribution with $df$ degrees of freedom
3. Compute the upper bound of a 95% confidence interval:
   $UB_{q}=\frac{s_{q}^{2}+w_{M}^{2}}{2\left( \tilde{r}_{q}+w_{M} \right)}\left( \chi_{\frac{2\left( \tilde{r}_{q}+w_{M} \right)^{2}}{s_{q}^{2}+w_{M}^{2}}}^{2} \right)^{-1}\left( .975 \right)$ where $w_{M}=\max\left\{ w_{a,s}/N_{a,s,q} \right\}$ and the max is taken over the age-sex groups $a,s$ in SES quintile $q$
4. Take a difference between the rates in the highest and lowest quintiles
$\hat{\Delta}= \tilde{r}_{5}-\tilde{r}_{1}$
5. Form a 95% confidence bound for this difference using a Normal approximation:
   $\hat{\Delta}\pm z_{.975}\sqrt{s_{5}^{2}+s_{1}^{2}}$ where $z_{.975}$ is the .975 quantile of a standard normal distribution ($\approx1.96)$.

Age-sex-standardized outcomes

For each **hip fracture sample**, we computed outcomes standardized to the age-sex distribution of the bottom 20% of SES of the whole jurisdiction.

1. Assign each person a value of SES (individual- or neighborhood-level measure)
2. For each region
   1. Identify quintiles of SES: bottom 20%, second 20%, third 20%, fourth 20%, top 20%
   2. To each person, assign a variable equal to their regional quintile of SES, $q_{i}\in\{1,\ldots,5\}$
3. For the whole jurisdiction, compute standardization weights: the age-sex distribution in the bottom 20% of SES
   $w_{a,s}=\frac{n_{a,s,1}}{\sum_{a,s} n_{a,s,1}}$ where $n_{a,s,q}$ is the number of people with hip fracture in age group $a$, sex group $s$, and income quintile $q$
4. Compute the raw outcome rates:
   $r_{a,s,q}=\frac{d_{a,s,q}}{n_{a,s,q}}$ where $d_{a,s,q}=\sum_{i} y_{i,a,s,q}$ for $y_{i,a,s,q}$, a binary outcome indicator for person $i$ in age group $a$, sex group $s$, and income quintile $q$ and $n_{a,s,q}$ is the corresponding number of people in the hip fracture sample
5. Multiply outcome rates by weights and sum over age-sex groups:
$\tilde{r}_{q}= \sum_{a,s} w_{a,s}r_{a,s,q}$
6. Report age-sex standardized outcomes in each SES quintile: $\tilde{r}_{q} for q\in\left\{ 1,\ldots5 \right\}$

The procedure for estimating the variance and forming confidence intervals is the same as above. This procedure works for all binary and count outcome types listed above.

1. For more information on Ontario Discharge Abstract Data please visit <https://data.ontario.ca/dataset/discharge-abstract-database-dad-ontario-hospitals> [↑](#footnote-ref-1)
2. For more information on Manitoba data please visit: <https://umanitoba.ca/manitoba-centre-for-health-policy/data-repository> [↑](#footnote-ref-2)
3. For more information on the Israel data from Clalit please see: <http://clalitresearch.org/about-us/our-data/> [↑](#footnote-ref-3)
4. An example of another paper using the same data is García-Gómez, P., van Kippersluis, H., O'Donnell, O., & van Doorslaer, E. (2013). Long Term and Spillover Effects of Health Shocks on Employment and Income. *The Journal of human resources*, *48*(4), 873–909. https://doi.org/10.1353/jhr.2013.0031 [↑](#footnote-ref-4)
5. [Statistics Netherlands (2022)](https://www.cbs.nl/nl-nl/onze-diensten/maatwerk-en-microdata/microdata-zelf-onderzoek-doen/aanvraag-toegang-microdata) provides detailed information on the variables and observations in these data sources, their representativity, and the procedure to get data access. [↑](#footnote-ref-5)
6. The procedures were classified using the CBV, CVV and ZA classifications. Procedure codes occurring in the cohort were translated with the “verrichtingenthesaurus” (<https://trex.dhd.nl/>). Based on the translation these three classifications were assigned. Similar approach was followed by Rabbe et al. (in press). [↑](#footnote-ref-6)
7. For more information on Taiwan’s NHI Data and Cause of Death Data please visit: <https://dep.mohw.gov.tw/dos/cp-5119-59201-113.html> (in Chinese) [↑](#footnote-ref-7)
8. For more information on US Medicare Part A Data please visit: https://www.cms.gov/Research-Statistics-Data-and-Systems/Statistics-Trends-and-Reports/MedicareFeeforSvcPartsAB/MEDPAR [↑](#footnote-ref-8)
9. Within a general framework similar to the US, countries were allowed to adapt coding schemes to fit local context and practice. [↑](#footnote-ref-9)
